# Sex-specific influences of APOEε4 genotype on hippocampal neurogenesis and progenitor cells in middle-aged rats

**DOI:** 10.1186/s13293-025-00694-8

**Published:** 2025-02-05

**Authors:** Bonnie H. Lee, Melike Cevizci, Stephanie E. Lieblich, Liisa A. M. Galea

**Affiliations:** 1 Graduate Program in Neuroscience, University of British Columbia, Vancouver, BC Canada; 2 Djavad Mowafaghian Centre for Brain Health, University of British Columbia, Vancouver, BC Canada; 3 Campbell Family Mental Health Research Institute, Centre for Addiction and Mental Health, Toronto, ON Canada; 4 Department of Psychology, University of British Columbia, Vancouver, BC Canada; 5 Department of Psychiatry, University of Toronto, Toronto, ON Canada

**Keywords:** Sex differences, Alzheimer’s disease, Hippocampus, Microglia, Male, Female

## Abstract

**Background:**

Alzheimer’s disease (AD) disproportionately and uniquely affects females, and these sex differences are further exacerbated by the presence of Apolipoprotein (APOE) ε4 alleles, the top genetic risk factor for late-onset AD. To expand our understanding about how late-onset AD risk might differentially influence males and females, this study explores how APOEε4 affects hippocampal neurogenesis and microglia, key neuroplastic markers involved in AD pathogenesis, differently by sex in middle-aged rats.

**Methods:**

A rat model expressing the humanized (h) APOEε4 allele was characterized to examine markers of adult neurogenesis (neural progenitor cells and new-born neurons) and immune cells (microglia) in the dentate gyrus of the hippocampus in 13 month-old male and female rats.

**Results:**

We observed basal sex differences in neurogenesis at middle age, as wildtype male rats had greater densities of neural progenitor cells and new-born neurons in the dentate gyrus than wildtype female rats. Male hAPOEε4 rats exhibited fewer neural progenitor cells, fewer new-born neurons, and more microglia than male wildtype rats. On the other hand, female hAPOEε4 rats exhibited more new-born neurons than female wildtype rats. Interestingly, females had more microglia than males regardless of genotype. Correlations were conducted to further elucidate any sex differences in the relationships between these biomarkers. Notably, there was a significant positive correlation between neural progenitor cells and new-born neurons, and a significant negative correlation between new-born neurons and microglia, but only in male rats.

**Conclusion:**

In contrast to the clear pattern of effects of the hAPOEε4 risk factor on hippocampal neurogenesis in males, females had unaltered levels of neural progenitor cells and increased density of new-born neurons. Furthermore, relationships between neurogenesis and microglia were significantly correlated within males, and not females. This suggests that females may be presenting a compensatory response to the hAPOEε4 genotype at middle age. Collectively, these results exemplify the importance of thoroughly examining influences of sex on AD endophenotypes, as it may reveal sex-specific pathways and protective mechanisms relevant to AD.

## Abstract (plain language summary)

Female sex and Apolipoprotein (*APOE*) ε4 genotype are top risk factors for late-onset Alzheimer’s disease. This research investigates how these two risk factors might interact to influence biomarkers of brain health at middle age using a rat model. Compared to healthy controls, male rats with hAPOEε4 genotype showed reduced neural stem cell-like cells and new adult-born brain cells and increased microglia (marker of inflammation in the brain) at middle age. In contrast, female rats with hAPOEε4 genotype showed increased new adult-born neurons, but no changes in the other cell types, suggesting a possible compensatory response to the effects of hAPOEε4 at this time point. These results highlight the importance of examining sex-specific pathways in AD, as they may uncover unique protective mechanisms and inform development of tailored treatment strategies.

## Highlights

In male middle-aged rats, hAPOEε4 genotype decreased adult neurogenesis on the level of both neural progenitor cells and new-born neurons and increased the density of microglia in the dentate gyrus.

In female middle-aged rats, hAPOEε4 genotype increased neurogenesis on the level of new-born neurons and did not significantly affect density of neural progenitor cells.

In male rats only, there was a significant positive correlation between neural progenitor cells and new-born neurons and a significant negative correlation between new-born neurons and microglia.

This research shows differential effects of hAPOEe4 genotype on hippocampal neurogenesis and microglia between males and females, underscoring the importance for future research to consider sex-specific influences on biomarkers and processes associated with AD.

Alzheimer’s disease (AD) is a progressive neurodegenerative disorder characterized by cognitive decline and pathological changes in the brain [1]. Over 90% of AD cases are sporadic, occurring after 65 years of age in humans [2]. In addition to advancing age, the greatest non-modifiable risk factors for sporadic AD are female sex and Apolipoprotein (*APOE*) ε4 genotype. Human females experience greater lifetime risk for sporadic AD, and those with AD have elevated levels of neuropathology and faster cognitive decline compared to human males with AD [3–6]. Apolipoprotein E (*APOE*) accounts for approximately 25% of the total heritability of sporadic AD; inheriting one or both copies of the APOE ε4 alleles increases risk by 3 or 15-fold, respectively, and at least one copy of the allele is present in 40–65% of individuals with AD [7]. Furthermore, APOEε4 genotype is associated with increased amyloid deposition and dysfunction of the medial temporal lobe, features related to AD [8–10]. Intriguingly, the combination of female sex and APOEε4 genotype presents with more endophenotypes of AD, as female APOEε4 carriers experience greater AD risk, AD neuropathology, including phosphorylated tau, and cognitive impairment compared to male APOEε4 carriers [11–15]. Understanding how the relationship between sex and APOEε4 genotype can contribute to AD manifestation and progression is key to informing precision medicine in AD. Furthermore, examination of brain health biomarkers at middle age is particularly relevant due to the increasing risk for late-onset AD during this period as well as heightened sex differences in the impact of APOEε4 on AD risk [16, 17].

The hippocampus is a particularly compelling brain region to investigate because it is one of the first regions impacted by the pathogenesis of AD and compromised hippocampal integrity is related to the decline in hippocampus-dependent cognitive functions in AD [18–21]. The dentate gyrus of the hippocampus retains its ability to produce new neurons throughout life in all mammalian species studied [22, 23]. Although there are some reports contesting the existence of newly generated neurons in the adult human hippocampus [24, 25], these findings have been challenged based on methodology [26–29]. Furthermore, there are multiple lines of evidence, derived from various methods, in support of hippocampal neurogenesis persisting in adult humans [22, 23, 30–37]. The process of neurogenesis involves neural progenitor cells that migrate, differentiate, and mature into neurons. These new-born neurons can modulate functions of the hippocampus, including pattern separation, which is compromised early in AD [38, 39]. Neurogenesis in the hippocampus is closely linked to the pathogenesis of AD [40] and altered neurogenesis might have important implications for the cognitive deficits seen in AD [41]. There is literature showing that hippocampal neurogenesis is increased early in the disease, potentially as a compensatory mechanism [42, 43], and decreased as AD severity advances in later stages [18, 40]. There are striking sex differences in the hippocampus and the trajectory and regulation of adult neurogenesis [44, 45]. In addition to morphological differences, the temporal dynamics of neurogenesis differ by sex, such that males have faster maturation and attrition rates of adult-born neurons compared to female rats [45]. Together, these findings indicate that hippocampal neurogenesis is intricately involved in the progression of AD, and thus, investigating how it might be differentially impacted by sex and APOEε4 genotype will expand our knowledge about hippocampal neurogenesis as an endophenotype of, and potential precision therapeutic target for, AD.

Inflammation in the brain is elevated with AD, and chronic activation of immune cells in the brain, such as microglia, plays a key role in the neurodegenerative cascade in AD [46, 47]. Microglia contribute to the construction and maintenance of neuronal networks in a healthy state, but can disrupt brain circuits and connectivity in AD [48]. Inflammation also impacts neuronal loss and neurogenesis in the hippocampus [48–51]. In terms of differences with aging, there are more microglia in the dentate gyrus of aged (20–24 month) and middle-aged (13 month) female rats compared to young adult female rats [52], and in the CA1 of aged (20–24 month old) female mice compared to both young (3–4 month old) female mice and aged male mice [53]. Single cell RNA sequencing data have further revealed greater sex differences in microglia from aged (22–25 month old) mice compared to young (5–6 month old) mice, and a stronger effect of aging on gene expression in female microglia compared to male microglia [54, 55]. Although beyond the scope of the present study, it is important to consider the contribution of declining or fluctuating levels of steroid hormones across the lifespan to changes in inflammatory processes, including microglia levels and phenotype diversity [56]. It is becoming increasingly evident that the relationships and dynamics between inflammation and other hallmarks of AD may differ across sex [57, 58]. There are relatively few studies investigating sex differences in inflammation in the context of AD to date, and the existing literature is mixed. For example, a study designed to understand the vascular and cognitive contributions to AD found increased activation of microglia with a high fat diet in male, but not female, middle-aged mice [57]. Another study found increased markers of inflammation, including microglia, and amyloid pathology in female compared to male 5XFAD 5-month old mice [59]. Although these findings seem equivocal, differences in age, model, and diet of animals used in the studies influence these effects and need to be considered. Analyses using a large human database identified osteoporosis as a female-specific clinical predictor of AD, with shared relationships through the gene *MS4A6A*, which is involved in immune function, particularly among microglia [60].

The objective of this study was to characterize sex differences in the influence of APOEε4 genotype on hippocampal neurogenesis and microglia in middle-aged rodents. Progenitor cells, new-born immature neurons, and microglia in the dentate gyrus were quantified and morphological states of microglia were assessed to examine their putative inflammatory profile in the hippocampus. We hypothesized that male and female rats will be distinctly impacted by APOEε4, displaying different neurogenesis levels and inflammatory phenotypes.

## Methods

### Subjects

Twenty-six Sprague Dawley rats were evenly divided into 4 groups; male and female rats were either wildtype or expressed (humanized (h) APOEε4 (HsdSage: SD-ApoEem1Sage rat, developed by SAGE Labs, Inc., Saint Louis, MO, USA). Males and females were housed in separate colony rooms that were maintained on a 12-hour light/dark cycle (lights on at 07:00 h) in standard laboratory conditions (21 ± 1 °C; 50 ± 10% humidity). All rats were given ad libitum access to food (Purina Rat Chow) and water. All experiments were conducted in accordance with ethical guidelines set by the Canada Council for Animal Care, and all procedures were approved by the University of British Columbia Animal Care Committee. All efforts were made to reduce the number and suffering of animals.

### Procedure and tissue collection

At middle age (13 months old), female rats were vaginally lavaged daily for 10 days until euthanasia. Lavage samples were transferred onto microscope slides, stained with Cresyl Violet, and left to dry. Lavage samples were qualitatively categorized as diestrus (consisting primarily of leukocyte-dense cells), proestrus (consisting primarily of nucleated epithelial cells), or estrus (consisting primarily of cornified cells), for evidence of irregular cycling, which was defined as consecutive lavage cycles varying in length and/or order. All rats were euthanized at 13 months old via lethal overdose of sodium pentobarbital. Brains were extracted and cut longitudinally into halves. The right hemispheres were flash frozen on dry ice and stored at − 80 °C, then sliced into 300 μm coronal sections at -10 °C using a cryostat (CM3050 S; Leica, Nuβloch, Germany). Punches of 1.25 mm were used to extract tissue from cortex (within bregma − 3.30 mm to -3.80 mm, in the area directly above the hippocampus). The tissue was then homogenized using an Omni Bead Ruptor (Omni International, Kennesaw, GA) with 120 µl of cold Tris lysis buffer. Homogenates were centrifuged at 1000xg for 5 min at 4 °C and supernatants were stored at -80 °C for later analysis (Sect. 2.6.1 and 2.6.2). The left hemispheres were post-fixed for 24 h in 4% paraformaldehyde (at 4 °C), then transferred to a 30% sucrose solution for cyroprotection until the brains sank. Using a freezing microtome (2M2000R; Leica, Richmond Hill, ON), the left hemisphere of each brain was sliced into 35 μm coronal sections and collected in series of 5 throughout the frontal cortex and in series of 10 throughout the entire rostral-caudal extent of the hippocampus. Sections were stored in a cryoprotective medium (consisting of 0.1 M PBS, 30% ethylene glycol, and 20% glycerol) at − 20 °C.

### Amyloid-beta peptide electrochemiluminescence assay

Aβ42/Aβ40 ratio in the cortex was measured as an indicator of amyloid accumulation in the brain. This ratio was calculated as it is considered a more reliable marker of brain amyloid production and its downstream effects compared to Aβ42 or Aβ40 levels alone [61–63]. Amyloid-beta (Aβ) peptide concentrations in cortex homogenates were measured using a 3-plex electrochemiluminescence immunoassay kit (V-PLEX Aβ Peptide Panel 1) from Meso Scale Discovery (Rockville, MD, USA) according to the manufacturer’s instructions. Samples were run in duplicates to quantify Aβ38, Aβ40, and Aβ42. Plates were read using a Sector Imager 2400 (Meso Scale Discovery), and data analyses were conducted using the Discovery Workbench 4.0 software (Meso Scale Discovery).

### Immunohistochemistry

All immunohistochemical procedures were conducted on free-floating brain sections and on a rotator at room temperature unless otherwise noted. After staining, sections were mounted onto glass slides, allowed to dry, then dehydrated in increasing graded ethanol, defatted with xylene, and cover-slipped with Permount (Fisher Scientific).

#### Sry-box transcription factor 2

Sox2 is critical for maintaining pluripotency and is considered a marker of neural progenitor cells [64]. One series of hippocampal sections was stained for SRY-box transcription factor 2 (Sox2). Tissue was thoroughly rinsed (3 × 10 min) in 0.1 M tris-buffered saline (TBS; pH 7.4) before staining and between each of the following procedures. Tissue was first treated with 3% hydrogen peroxide (H2O2 in dH2O) for 30 min, then blocked with TBS + solution containing 3% normal horse serum and 0.3% Triton-X in 0.1 M TBS for 30 min. Tissue was incubated in a primary antibody solution containing 1:1000 mouse anti-Sox2 (Santa Cruz Biotechnology, Santa Cruz, CA, USA) in TBS + for 48 h at 4 °C. Next, tissue was incubated in a secondary solution containing 1:2 ImmPRESS^®^ (peroxidase) polymer horse anti-mouse IgG (rat absorbed) (Vector Laboratories) in TBS for 30 min. This ImmPRESS polymerized reporter enzyme staining system was used to enhance detection of mouse primary antibodies on rat tissues that may contain endogenous rat immunoglobulins. Immunoreactants were visualized with a Nickel-enhanced DAB reaction (Vector Laboratories).

#### Doublecortin

One series of hippocampal sections was stained for doublecortin (DCX), a microtubule-associated protein expressed in new-born immature neurons [65]. Tissue was thoroughly rinsed (3 × 10 min) in 0.1 M phosphate-buffered saline (PBS; pH 7.4) before staining and between each of the following procedures. Tissue was first treated with 0.6% hydrogen peroxide (H2O2 in dH2O) for 30 min, then incubated in a primary antibody solution containing 1:1000 goat anti-doublecortin (Santa Cruz Biotechnology, Santa Cruz, CA, USA) in 3% normal rabbit serum and 0.4% Triton-X in 0.1 M PBS for 24 h at 4 °C. Next, tissue was incubated in a secondary antibody solution containing 1:500 biotinylated rabbit anti-goat (Vector Laboratories, Burlington, ON, Canada) in 0.1 M PBS for 24 h at 4 °C. Lastly, tissue was transferred to an avidin–biotin complex (Elite kit; 1:1000, Vector Laboratories) for 4 h. Immunoreactants were visualized with a Nickel-enhanced DAB reaction (Vector Laboratories).

#### Ionized calcium-binding adaptor molecule 1

One series of hippocampal sections was stained for ionized calcium-binding adaptor molecule 1 (Iba1), a marker of microglia [66]. Tissue was thoroughly rinsed (3 × 10 min) in 0.1 M TBS (pH 7.4) before staining and between each of the following procedures. Tissue was treated with 3% hydrogen peroxide (H_2_O_2_ in dH_2_O) for 30 min, then blocked with TBS + solution containing 3% normal donkey serum and 0.3% Triton-X in 0.1 M TBS for 30 min. Tissue was incubated in a primary antibody solution containing 1:1000 rabbit anti-Iba1 (Wako, Osaka, Japan) in TBS + for 48 h at 4 °C. Next, tissue was incubated in a secondary antibody solution containing 1:250 donkey anti-rabbit Alexa Fluor 594 (Vector Laboratories) in TBS + for 4 h. Lastly, tissue was incubated in 1:1000 DAPI in TBS for 2.5 min, then mounted onto slides and cover-slipped with PVA DABCO.

### Microscopy and cell quantification

Cell quantification was conducted while blinded to experimental conditions of animals. Brain regions were defined according to a standard rat brain atlas [67]. Density of immunoreactive (IR) cells was calculated by dividing the total number of cells by area (µm^2^) of the corresponding region. The dorsal hippocampus was located in Sect. 7.20 to 4.48 mm from the interaural line (Bregma − 1.80 to -4.52 mm and the ventral hippocampus was located in Sect. 4.48 to 2.20 mm from the interaural line (Bregma − 4.52 to -6.80 mm). As these subregions of the hippocampus possess different functional capabilities [68] and exhibit different timelines of neuronal maturation [45], cells were quantified from 2 sections of dorsal dentate gyrus and 2 sections of ventral dentate gyrus. Sections stained for Sox2 were imaged using the Zeiss Axio Scan.Z1 (Carl Zeiss Microscopy, Thornwood, NY, USA) with a 20x objective lens and brightfield imaging. Sox2-immunoreactive (IR) cells in the granule cell layer of the dentate gyrus were counted using a MATLAB (MathWorks) code developed by JEJS and modified by NT as described in Yagi et al. (2020). This code can be made available by contacting the corresponding author. Briefly, images were converted to 8-bit, binarized, and thresholded. Size restrictions were applied to remove artifacts that are too large (bigger than 500 pixels) or too small (smaller than 10 pixels) to be a cell. The number of times the background is removed from the image is adjusted and optimized for the stain.

Sections stained for DCX were viewed under a 100x objective on an Olympus CX22LED brightfield microscope, and DCX-IR cells in the granule cell layer of the dentate gyrus were exhaustively counted. Area measures of the regions of interest were obtained using images acquired with the Zeiss Axio Scan.Z1 (Carl Zeiss Microscopy, Thornwood, NY, USA) with a 10x objective lens and brightfield imaging.

Sections stained for Iba1 were imaged with Zeiss Axio Scan 7 (Carl Zeiss Microscopy, Thornwood, NY, USA) with a 40x objective lens using fluorescent light. Iba1-IR cells were counted from images of 2 sections each from the dorsal and ventral dentate gyrus using ImageJ. As microglia assume different morphological phenotypes in response to surrounding conditions, a random sampling of Iba1-IR cells were categorized by morphology [69, 70]. A macro on ImageJ was run to randomly select 20 Iba1-IR cells within the region of interest, defined from two sections each of dorsal and ventral dentate gyrus. Each Iba1-IR cell was then manually classified as ramified (long, highly branched processes to survey the environment under homeostatic conditions), stout (fewer and shorter processes), or ameboid (enlarged and rounded cell body, no processes, typically seen under inflammatory conditions).

### Statistical analyses

Analyses were conducted using Statistica (Statsoft Tulsa, OK). Repeated measures analysis of variance (ANOVA) were used on dependent variables of interest with genotype (wildtype, hAPOEε4) and sex (male, female) as between-subjects variables and region (dorsal dentate gyrus, ventral dentate gyrus) as within-subjects variables. Post-hoc tests used Newman-Keuls and any a priori comparisons to examine sex by genotype interactions were subjected to Bonferroni correction. A Chi-square test was used to compare the frequency of rats that displayed irregular estrous cycling and constant estrous cycling. Estrous cycle phase was used as a covariate in all analyses but did not change any outcomes.

## Results

### Wildtype males had a higher density of Sox2-IR cells in the dentate gyrus compared to hAPOEε4 males and wildtype females

**Fig. 1:**
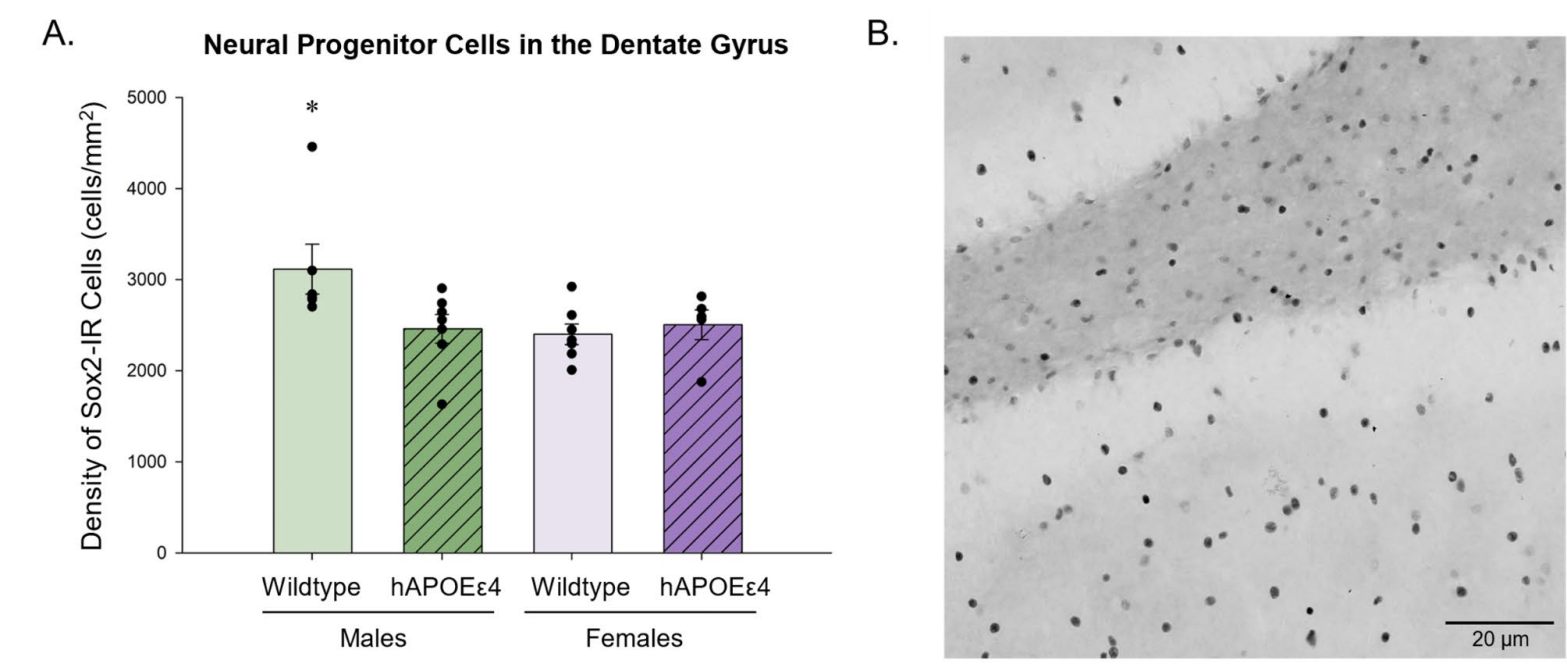
(**A**) Average density of Sox2-IR cells ± standard error of the mean in the dentate gyrus. Wildtype males had a greater density of Sox2-IR cells than all other groups in the dentate gyrus. There was a greater density of Sox2-IR cells in the ventral dentate gyrus than the dorsal dentate gyrus. (**B**) Photomicrograph of Sox2-IR cells. * indicates *p* < 0.05. Sox2– SRY-box transcription factor 2, DG– dentate gyrus, hAPOEε4– humanized APOEε4

Sox2-IR cells were quantified to examine potential differences in the density of neural progenitor cells in the dentate gyrus. Wildtype males had a higher density of Sox2-IR cells in the dentate gyrus compared to all other groups (hAPOEε4 males (*p* = 0.050, Cohen’s *d* = 1.278; wildtype females (*p* = 0.029, Cohen’s *d* = 1.499; hAPOEε4 females (*p* = 0.055, Cohen’s *d* = 1.240; sex by genotype interaction: *F* [1, 21] = 4.240, *p* = 0.050, partial η^2^ = 0.168; Fig. 1), but there was no statistical difference between wildtype and hAPOEε4 females (*p* = 0.701, Cohen’s *d* = 0.343). There was a higher density of Sox2-IR cells in the ventral dentate gyrus (1430.924 ± 79.477) than the dorsal dentate gyrus (1178.724 ± 43.156; main effect of region: *F* [1, 19] = 12.104, *p* = 0.002, partial η^2^ = 0.366; Fig. 1), but no other significant main effects or interactions (all p’s > 0.08).

### Wildtype males had a higher density of DCX-IR cells in the dentate gyrus than hAPOEε4 males, whereas the opposite pattern was seen in females

**Fig. 2:**
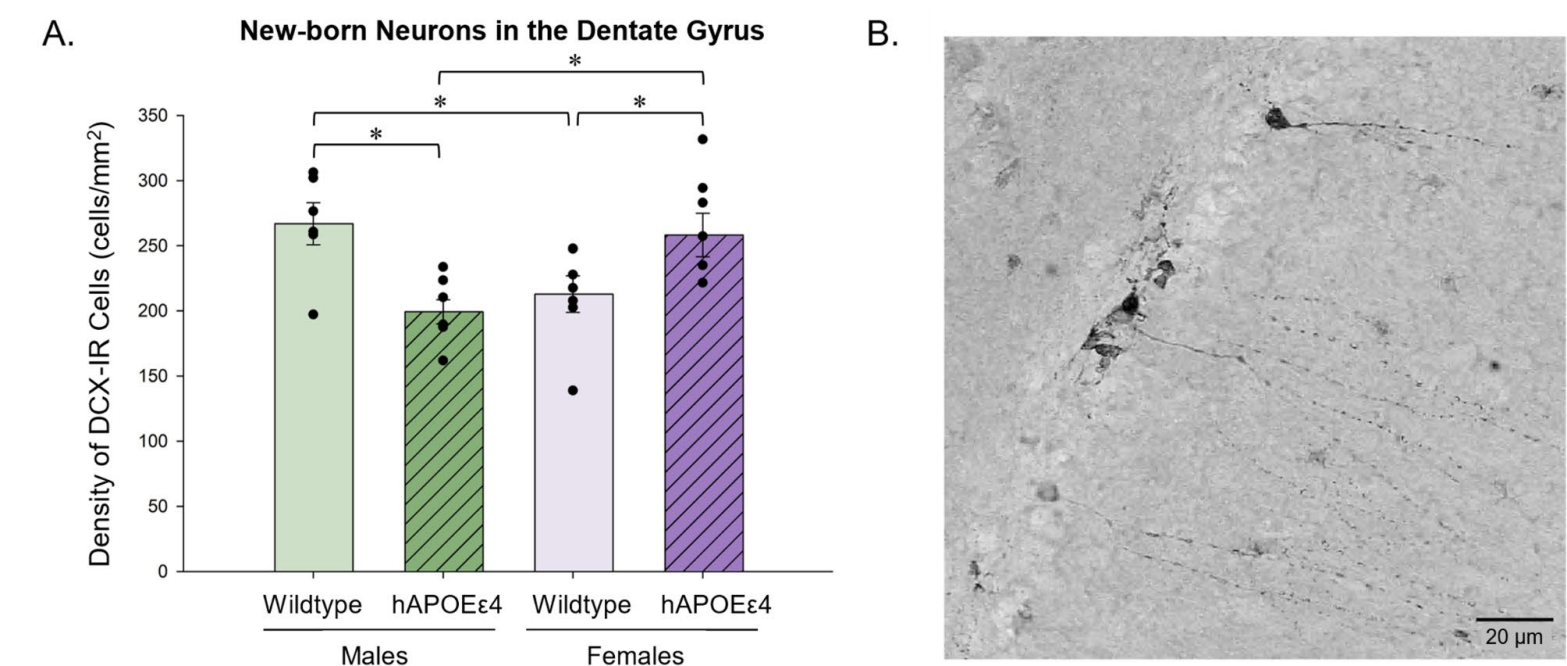
(**A**) Average density of DCX-IR cells ± standard error of the mean in the dentate gyrus. hAPOEε4 males had a lower density of DCX-IR cells than wildtype males, whereas hAPOEε4 females had a greater density of DCX-IR cells than wildtype females. (**B**) Photomicrograph of DCX-IR cells. * indicates *p* < 0.05. DCX– doublecortin, DG– dentate gyrus, hAPOEε4– humanized APOEε4

DCX-IR cells were quantified to examine potential differences in the density of new-born immature neurons in the dentate gyrus. Wildtype males had a higher density of DCX-IR cells in the dentate gyrus compared to hAPOEε4 males (*p* = 0.007, Cohen’s *d* = 2.236) and compared to wildtype females (*p* = 0.013, Cohen’s *d* = 1.530; sex by genotype interaction *F* [1, 22] = 19.831, *p* < 0.001, partial η^2^ = 0.474; Fig. 2). However, an opposing pattern was seen in females, as wildtype females had a lower density of DCX-IR cells than hAPOEε4 females (*p* = 0.022, Cohen’s *d* = 1.453; Fig. 2). hAPOEε4 female rats also had greater density of DCX-IR cells than hAPOEε4 males (*p* = 0.008, Cohen’s *d* = 2.328; Fig. 2). There were no other significant main effects or interactions (all p’s > 0.189).

### hAPOEε4 rats had a greater density of Iba1-IR cells in the dentate gyrus than wildtype rats. Female rats had a greater density of Iba1-IR cells in the dentate gyrus than male rats

**Fig. 3:**
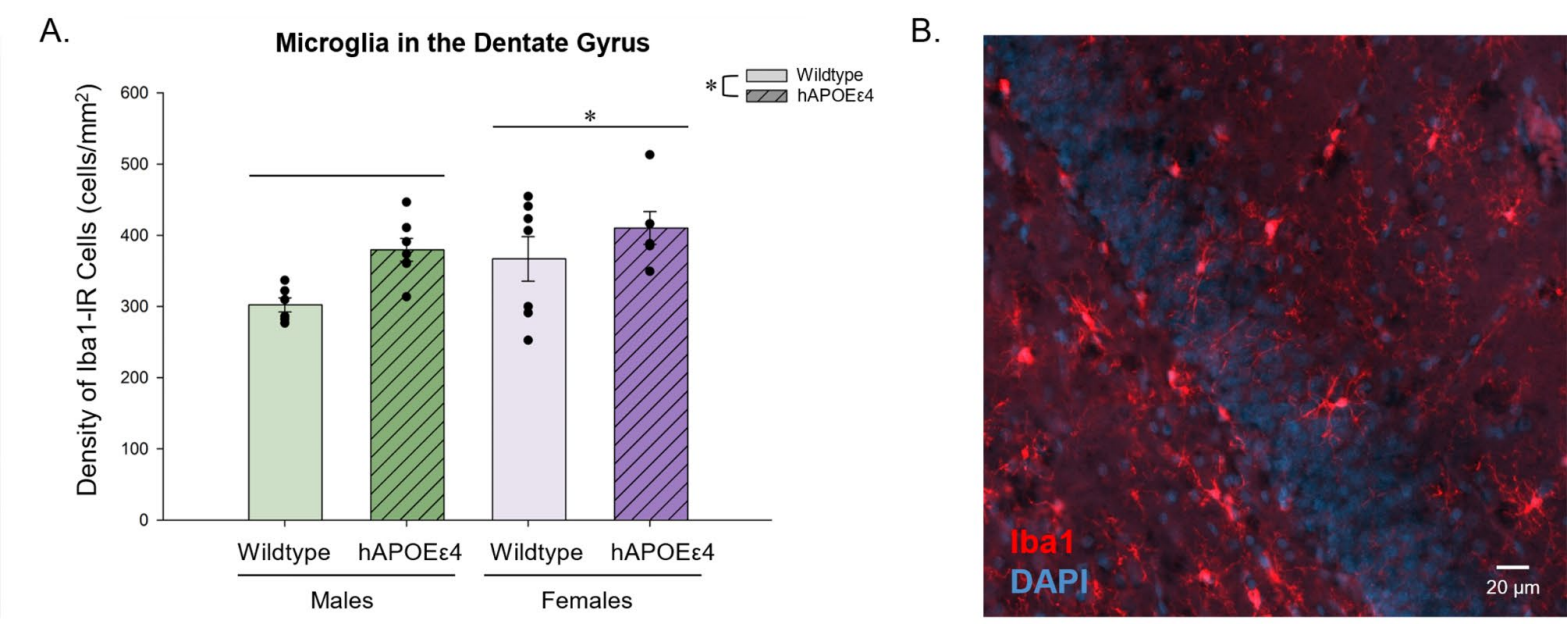
Average density of Iba1-IR cells ± standard error of the mean in the dentate gyrus. hAPOEε4 rats had a higher density of Iba1-IR cells than wildtype rats. Female rats had a higher density of Iba1-IR cells in the dentate gyrus than male rats. (**B**) Photomicrograph of Iba1-IR cells. * indicates *p* < 0.05. Iba1– ionized calcium-binding adaptor molecule 1, DG– dentate gyrus, hAPOEε4– humanized APOEε4

Iba1-IR cells were quantified to examine potential differences in the density of microglia in the dentate gyrus. hAPOEε4 rats had a higher density of Iba1-IR cells, regardless of region compared to wildtype rats (main effect of genotype: *F* [1, 22] = 6.898, *p* = 0.015, partial η^2^ = 0.015; Fig. 3). There was also a main effect of sex such that females had a higher density of Iba1-IR cells compared to males (*F* [1, 22] = 4.223, *p* = 0.050, partial η^2^ = 0.161; Fig. 3). There were no other significant main effects or interactions (all p’s > 0.228).

### There was a greater proportion of ramified Iba1-IR cells in the dorsal dentate gyrus than the ventral dentate gyrus

**Fig. 4:**
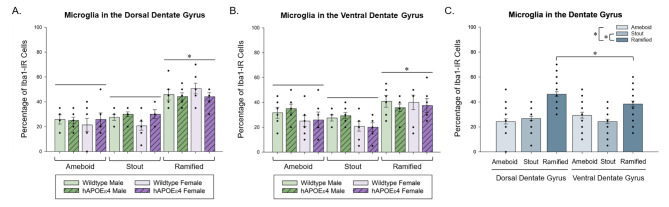
Total proportions of ameboid, stout, and ramified Iba1-IR cells ± standard error of the mean in the (**A**) dorsal and (**B**) ventral dentate gyrus. (**C**) Total proportions of ameboid, stout, and ramified Iba1-IR cells ± standard error of the mean in the dentate gyrus. There were more ramified than ameboid or stout Iba1-IR cells in the dentate gyrus, regardless of region. There were more ramified Iba1-IR cells in the dorsal dentate gyrus than the ventral dentate gyrus. * indicates *p* < 0.05. Iba1– ionized calcium-binding adaptor molecule 1, DG– dentate gyrus, hAPOEε4– humanized APOEε4

To assess potential differences in microglia morphological profiles, Iba1-IR cells in the dentate gyrus were classified as ameboid, stout, or ramified. There was a greater proportion of ramified Iba1-IR cells in the dorsal dentate gyrus compared to the ventral dentate gyrus (*p* = 0.014, Cohen’s *d* = 38.462; region by morphology type interaction: *F* [2, 44] = 4.254, *p* = 0.020, partial η^2^ = 0.162; Fig. 4). There was a main effect of type such that a greater proportion of Iba1-IR cells were ramified compared to ameboid or stout (main effect of type: *F* [2, 44] = 34.468, *p* < 0.001, partial η^2^ = 0.610; Fig. 4), but no other significant main effects or interactions (all *p*’s > 0.054).

### In male rats only, there was a significant positive correlation between density of Sox2-IR cells and DCX-IR cells and a significant negative correlation between density of DCX-IR cells and Iba1-IR cells in the dentate gyrus

**Fig. 5:**
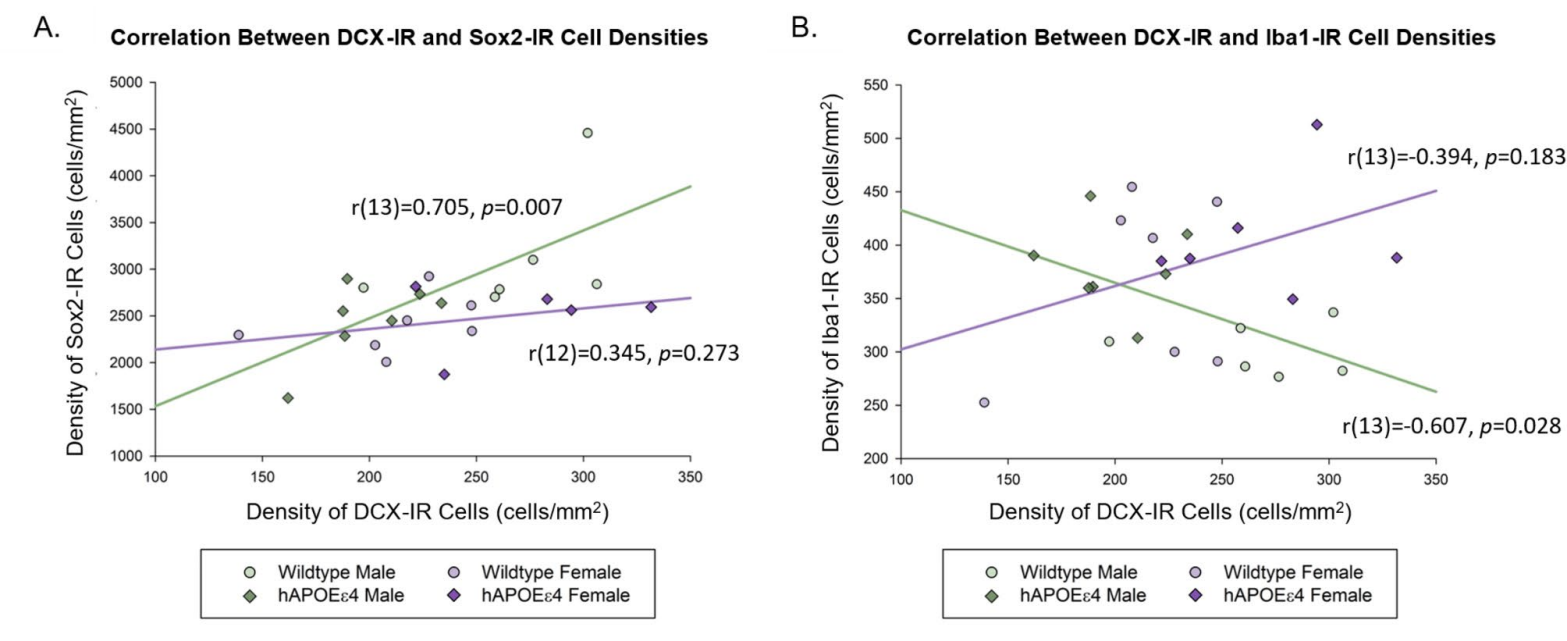
Scatterplots of correlations between (**A**) Sox2-IR and DCX-IR cell density and (**B**) Iba1-IR and DCX-IR cell density in male and female rats. There was a significant positive correlation between Sox2-IR and DCX-IR cell density and a significant negative correlation between Iba1-IR and DCX-IR cell density in male rats only. Sox2– SRY-box transcription factor 2, DCX– doublecortin, Iba1– ionized calcium-binding adaptor molecule 1, DG– dentate gyrus, hAPOEε4– humanized APOEε4

Pearson product-moment correlations were calculated between markers of neurogenesis and microglia. There was a significant positive correlation between density of Sox2-IR cells and density of DCX-IR cells in male (r [13] = 0.705, *p* = 0.007), but not female, rats (r [12] = 0.345, *p* = 0.273; Fig. 5). There was also a significant negative correlation between density of DCX-IR cells and density of Iba1-IR cells in male (r [13]=-0.607, *p* = 0.028), but not female, rats (r [13]=-0.394, *p* = 0.183; Fig. 5).

### Female APOEε4 rats had greater Aβ42/Aβ40 than female wildtype rats. Female rats had greater Aβ42/Aβ40 than male rats

**Fig. 6:**
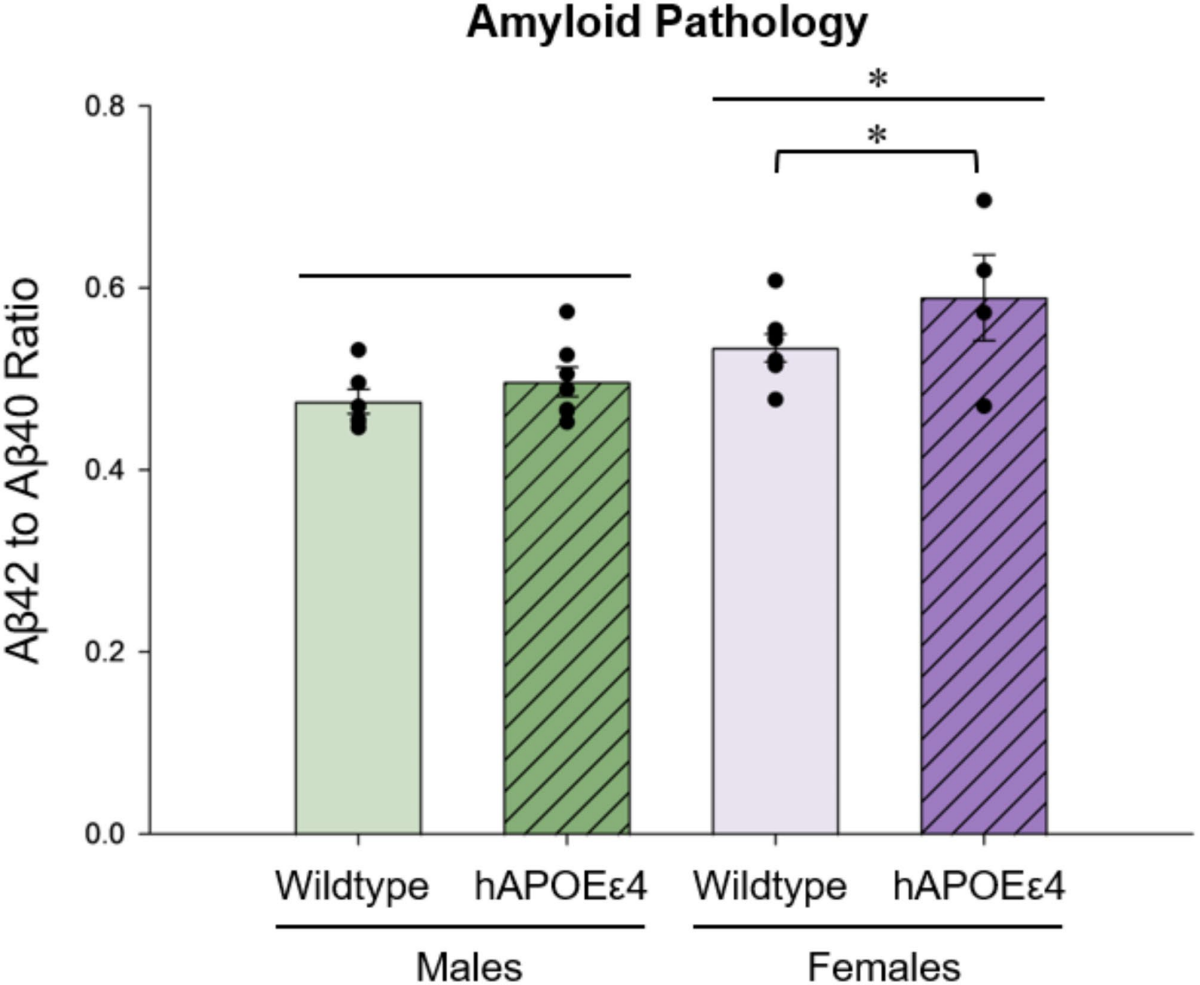
Mean Aβ42 to Aβ40 ratio in the cortex. Female APOEε4 rats showed a higher ratio, which indicates greater amyloid pathology, compared to female wildtype rats. Female rats also showed a higher ratio compared to male rats. Aβ– amyloid beta, hAPOEε4– humanized APOEε4

Ratio of Aβ42 to Aβ40 in the cortex was calculated to examine potential effects of APOEε4 genotype and sex on amyloid pathology. Planned comparisons revealed that female APOEε4 rats had higher Aβ42/Aβ40 compared to female wildtype rats (one-tailed *p* = 0.0451, Cohen’s *d* = 0.761; sex by genotype interaction: *F* [1, 20] = 0.619, *p* = 0.440, partial η^2^ = 0.030; Fig. 6). There was also a main effect of sex such that female rats had higher Aβ42/Aβ40 compared to male rats (*F* [1, 20] = 12.296, *p* = 0.002, partial η^2^ = 0.381; Fig. 6).

### Estrous cycling of female rats did not alter any findings

Of the female rats in this study, 77% displayed a constant estrous stage and the remaining 23% displayed irregular estrous cycling. There were no genotype differences in proportion of rats displaying constant estrous stage or irregular estrous cycling (*p* = 0.416). Using estrous cycling (classified as irregular or in constant estrus) as a covariate did not alter any findings, nor was there a significant effect of the covariate (all *p*’s > 0.142).

## Discussion

The findings of the present study demonstrate striking sex differences in hippocampal neurogenesis at middle age that are dependent on hAPOEε4 genotype. Specifically, hAPOEε4 males exhibited fewer neural progenitor cells and fewer new-born neurons compared to wildtype males. On the other hand, hAPOEε4 females showed no changes in density of neural progenitor cells and more new-born neurons compared to wildtype females. hAPOEε4 rats had elevated levels of microglia than wildtype rats and females, regardless of genotype, had elevated levels of microglia compared to males. Significant relationships between neurogenesis and neural progenitor cells or microglia were present in male, but not female, rats. Together, these findings highlight sex differences in signatures relating to neurogenesis and microglia in a model of AD risk, underscoring the need to develop and apply sex-specific approaches within AD research and treatment development.

### hAPOEε4 genotype reduced the density of neural progenitor cells in males but not females

Perhaps as expected, given the increased risk for AD with APOEε4 genotype, hAPOEε4 male rats had fewer neural progenitor cells (Sox2-IR) in the dentate gyrus relative to wildtype rats. However, the same pattern was not detected in female rats, as there were no significant effects of genotype on neural progenitor cells in the dentate gyrus in females. In individuals with AD, Sox2 is decreased in the brain and there is functional crosstalk between Sox2 and proteins involved in AD [71]. Thus, neural stem or progenitor cell density may be a protective factor for AD. Indeed, a meta-analysis indicated that neural stem cell therapy improved cognition and reduced levels of neuropathology in preclinical AD models [72]. However, 80% of the 30 studies included in the meta-analysis were either exclusively performed in males or did not report the sex of animals used, and only 3 studies examined females alone [72]. Our findings have implications for sex differences in treatment efficacy of neural progenitor cell therapy for AD– at least for those with APOEε4 genotype– as the compromised density of neural progenitor cells in the dentate gyrus was only seen in hAPOE4 males and not females.

We also found that wildtype male rats had a greater density of neural progenitor cells than wildtype female rats at middle age. This aligns with previous work showing greater density of Sox2-IR cells in the dorsal dentate gyrus of male compared with female two-month-old rats [45]. It is interesting to note that Yagi et al. (2020) only found this sex difference in the dorsal dentate gyrus, whereas the present study found this sex difference in both the dorsal and ventral dentate gyrus. This could be attributed to the age difference in animals used in the studies, as age might modulate the effects of sex on density of neural progenitor cells in the dentate gyrus.

### hAPOEε4 genotype decreased neurogenesis in males, but increased neurogenesis in females

In line with the neural progenitor cell data (Sect. 3.1), we found that male hAPOEε4 rats also exhibited fewer new-born neurons compared to wildtype rats. In contrast, female hAPOEε4 rats exhibited more new-born neurons in the dentate gyrus than wildtype rats. This reduction, in both neural progenitor cells and new-born immature neurons, observed in males with hAPOEε4 genotype suggests that hAPOEε4 reduces neuroplasticity in the hippocampus by disrupting both the neural progenitor pool and generation of new-born neurons. This finding is consistent with research showing lower levels of neurogenesis in humans with AD compared to healthy controls [36, 40] as well as in various animal models of AD [73–76]. Both male and female 3xTg-AD mice show greater reduction in neurogenesis compared to controls, although female mice showed an earlier reduction at 4 months old and male mice showed the reduction at 9 months old [75]. However, it is important to consider the contribution of the various gene mutations involved in such transgenic animal models when interpreting findings and comparing across studies. Here, we focused on characterizing the effects of hAPOEε4 as it is the greatest known genetic risk factor for late-onset AD [7]. The present study starkly demonstrates that the effects of hAPOEε4 genotype on neurogenesis is very different in females. The increase in neurogenesis with hAPOEε4 genotype may be a compensatory mechanism in females at early stages of disease. This idea is supported by other studies– one conducted in female mice and three that did not detail sex of animals used or analyses by sex– that similarly showed increased hippocampal neuroplasticity and neurogenesis in early AD [42, 43, 77, 78]. It is important to note that despite an increase in neurogenesis, the newly generated neurons may not fully mature or may not develop into the right type of neurons that functionally contribute to circuitry as expected. Indeed, a study examining middle-aged female rats similarly found increased levels of neurogenesis in the hippocampus of hAPOEε4 rats compared to wildtype rats. Despite this, hAPOEε4 rats made more spatial working memory errors than wildtype rats, but no significant differences in activation of new-born neurons in response to memory retrieval between hAPOEε4 and wildtype female rats, suggesting the new-born neurons did not necessarily contribute to enhancing spatial working memory performance [79]. Future experiments should explore the survival and integration new-born neurons as well as how activity of new-born neurons might correlate with neural activity in other brain regions to explore changes with sex and hAPOEε4 at middle age on a network level. Nonetheless, the findings in females showing a “protective” effect on neuroplasticity in the hippocampus are reflected in human data, as human females, but not human males, had greater hippocampal integrity even in the face of amyloid pathology in midlife [80]. These results indicate that females may have a hippocampal reserve that is more resilient against early stages of the disease. In sum, the contrasting effects of hAPOEε4 on neural progenitor cells and new-born neurons in males and females in the present study suggest sex-specific responses to the presence of this AD risk factor, highlighting a key consideration for future research.

In wildtype rats, males had greater density of new-born neurons than females. This is in line with other studies reporting higher levels of cell proliferation in young adult male rats compared to female rats [45, 81]. It is possible that females have faster turnover of progenitor cells to proliferating cells, given the lack of change in progenitor cell density but increase in new-born neuron density. It is also possible that females have enhanced maturation of new-born neurons, however a previous study in younger rats found the opposite finding [45]. Future experiments to supplement these findings and elucidate sex differences in the trajectory of neurogenesis should use additional markers, including exogenous markers, to assess additional stages of neurogenesis. In addition, sex hormones can differentially modulate hippocampal neurogenesis in males and females, and age-related changes to steroid hormone levels and hormone receptor levels and reactivity in males and females can influence hippocampal neurogenesis in age-specific ways [82–84]. In the present study, we found that majority of female rats displayed a constant estrous stage, with the rest displaying irregular estrous cycling. This is in line with other research showing increased irregular estrous cycling with increased age in rats [52, 85]. Notably, irregularities in estrous cycle length followed by acyclicity are typical patterns of aging in rodents, leading up to cessation of hormone cycling and thus reproductive senescence [86, 87]. Although estrous cycle staging did not appear to influence our findings in the present study, it will be important for future research to more thoroughly assess whether changes to estrous cycling and steroid hormone levels might influence other biomarkers of neuroplasticity, as well as characterize these relationships at additional time points along the aging trajectory.

### hAPOEε4 genotype increased the density of microglia. Female rats had greater density of microglia compared to male rats

The present study found that hAPOEε4 genotype increased the density of microglia in the dentate gyrus, regardless of sex. This finding is somewhat inconsistent with research using a mouse model of vascular contributions to cognitive impairment and dementia, which reported increased activation of hippocampal microglia in male, but not female, middle-aged mice with a high fat diet [57]. However, the increase in microglia density in the present study was stronger in males (*p* = 0.008, Cohen’s *d* = 2.441) than in females (*p* = 0.151, Cohen’s *d* = 0.647; sex by genotype interaction: *F* [1, 22] = 0.717, *p* = 0.406, partial η^2^ = 0.032). This indicates differential pathways for AD risk depending on sex, and potentially that females may be exhibiting a compensatory response to hAPOEε4 genotype at middle age.

Females had more microglia in the dentate gyrus compared to males. Recent studies have shown sex differences in microglia and their involvement in AD pathways [58, 60, 88]. A large-scale meta-analysis of DNA methylation differences revealed sex-specific biological processes that affected AD neuropathology, including integrin activation and macrophage migration in females and complement activation in males [88]. Importantly, Zhang et al. demonstrate that many genes and biological processes previously implicated in AD neuropathology are predominantly driven by effects in only one sex, underscoring different regulatory mechanisms involved in AD neuropathology in males and females [88]. Our findings add to this literature and provide a foundation for future research to explore other inflammatory signatures including cytokines and the complement system.

Microglia assume diverse morphological characteristics in response to various stimuli. Generally, microglia exhibit a ramified morphology when surveilling their surroundings, including neighbouring neurons and other cells, whereas a transition towards an ameboid morphology signals a shift in microglial activation, often with increased phagocytic and migratory capacity [69]. In the present study, there were more ramified microglia in the dorsal dentate gyrus than the ventral dentate gyrus, and there were more ramified microglia than ameboid or stout microglia overall. This suggests that the activation and phagocytic potential of microglia might differ across subregions of the dentate gyrus. However, there were no significant effects of sex or genotype on the number of ameboid, stout, or ramified microglia in the dentate gyrus. This finding is perhaps unexpected, given that age-related changes in inflammatory and complement gene expression in the hippocampus occur earlier and to a larger extent in females compared to males [89]. Moreover, one study using a microglial transcript isolation approach identified a central APOE-driven network and found that shared microglial transcripts with this APOE network were exacerbated in females [90]. It is note-worthy that majority of sex differences reported in this study was in 24-month-old mice, despite also examining 3- and 12-month old mice. As such, we hypothesize that a more in-depth analysis of the different features related to microglial morphology and assessments at additional ages may further reveal insights to potential contributions of sex or hAPOEε4 genotype on microglia morphology.

### In male rats only, a greater density of new-born neurons was associated with a greater density of neural progenitor cells and a smaller density of microglia

We found a significant positive correlation between density of neural progenitor cells and density of new-born neurons in the dentate gyrus in males. We also found a significant negative correlation between density of new-born neurons and microglia in the dentate gyrus in males. These relationships were not seen in females, implicating that shifts in neurogenesis and microglia levels occur in tandem specifically in male, but not female, rats across both wildtype and hAPOEε4 groups. These findings emphasize the need for more research on how neurogenesis and inflammation interact in a sex-specific manner within AD risk models. As our current understanding about relationships between neurogenesis, inflammation, and AD are largely derived from male-only studies or research that has overlooked the influences of sex, addressing this gap is crucial for expanding our knowledge about AD and, ultimately, developing effective and tailored treatment options.

### Female hAPOEε4 rats showed greater amyloid pathology compared to female wildtype rats

We found that in females, hAPOEε4 rats had greater amyloid burden in the cortex, quantified as Aβ42/Aβ40, compared to wildtype rats. This is consistent with previous work in female rats that found increased Aβ42/Aβ40 as well as more errors in a spatial working memory task with hAPOEε4 genotype at middle age [79]. In the present study, we also found that females had greater amyloid burden compared to males. Interestingly, there were no genotype differences within male rats, suggesting that changes in neuroplasticity in the brain precedes amyloid-related pathology accumulation in male hAPOEε4 rats, and emphasizes there are sex differences in the effects of hAPOEε4 on biomarkers of brain health in middle-aged rats. Indeed, work by Caldwell et al. (2017) shows that despite greater amyloid burden in human females, hippocampal integrity remained remarkably intact, which is in line with what the present study shows in female rats [80]. Together, this underscores the importance of recognizing that pathways in aging and disease progression may not be similar across sexes. It will be interesting for future studies to characterize additional biomarkers related to AD pathology in males and females and understand the time course of each. Furthermore, it is important to acknowledge that the right hemisphere was used for the amyloid chemiluminescence assay whereas the left hemisphere was used for immunohistochemistry, and hemispheres were not counterbalanced. This was done to ensure consistency and control for any potential effects of laterality, although future studies should investigate whether there are any hemispheric differences in neuroplasticity and amyloid pathology.

### Perspectives and significance

Understanding the role of neuroplasticity in the onset and progression of AD is crucial to elucidating specific cellular contributions to the complex and multifaceted makeup of the disease. The present research revealed that hAPOEε4 genotype altered hippocampal neuroplasticity in male middle-aged rats in a manner that align with the “typical” effects hypothesized to be related to AD as reported in the literature. Importantly, these patterns may reflect the long-standing male bias embedded within research frameworks. Indeed, this study highlights biomarkers that are altered with hAPOEε4 in starkly different ways between males and females, which could indicate divergent downstream processes or even distinct mechanisms and timing of adaptation to AD risk in middle-aged male and female rats. Our findings align with work in humans that indicate females show more integrity in the face of greater amyloid pathology, specifically in the hippocampus, compared to males. Collectively, the findings of this work emphasize the need to rigorously consider and investigate sex as a critical factor in influencing aging and neurodegenerative disease pathways.

## Conclusion

Here, we found that hAPOEε4 genotype had opposing effects on hippocampal neurogenesis and inflammation depending on sex at middle age. Considering that presence of APOE ε4 alleles and female sex are top non-modifiable risk factors for AD, it is critical to understand how the interplay between these two factors might contribute to brain health. The present study showed that males hAPOEε4 rats exhibited lower density of neural progenitor cells and new-born neurons, as well as greater density of microglia, compared to male wildtype rats. In contrast, female hAPOEε4 rats exhibited greater density of new-born neurons, with minimal changes to neural progenitor cells and microglia, compared to female wildtype rats. Regardless of genotype, female rats had greater density of microglia compared to male rats. Future work should expand our understanding of how sex and hAPOEε4 genotype impact brain health by exploring additional stages of neurogenesis, along with a broader range of neuroplasticity and inflammatory biomarkers. Put together, our findings underscore the need to reassess existing knowledge about hippocampal neurogenesis and inflammation, and more broadly, brain health, in the context of AD, as it is largely based on studies using human males or male animal models. Accounting for influences of sex on the various endophenotypes of AD in future work will be key to informing targeted and effective treatments.

## Data Availability

Data available upon request to LAMG.
